# Rhode Island

**Published:** 1896-05

**Authors:** 


*Rhode Island.* Dr. F. H. Osgood, of the Massachusetts Cattle Commission, speaking on the subject of legislation at the recent convention of the various Commissioners of the

New England States, at Providence, said there was great need of inter-State laws regulating the traffic in animals in those States bordering on Massachusetts. Within their own States they had ample laws and machinery, but on the borders, where a great deal of traffic was involved, it was impossible to maintain a cordon of police to prevent diseased animals from slipping in, and the temptation to bring them in was great during 1894–95, when the State paid full value on a sound basis. The changes in the laws governing the Commission had worked no great injury to the value of their work, it had changed the plan and afforded more time for the education of those who opposed the use of tuberculin for diagnostic purposes, but these were being won over faster than the Commission could perform the work requested. The one great need to-day was the regulation of the sale of milk from other States within her borders. This should not be allowed unless the herds had been tested with tuberculin, and their freedom from disease certified to by competent veterinary inspectors, either of the adjoining States or delegated by Massachusetts to perform this work in the interest of her people. Ultimately, he thought, every herd would be examined by request or consent.

Veterinarian F. A. Rich, of the Vermont Experimental Station, speaking on the subject of the relation between bovine and human tuberculosis, said how different was the conception of this question some years ago, all of which was changed now, and how rapidly the people were appreciating the danger in an unguarded milk- and meat-supply. How much greater the need of watchfulness over the milk so largely used as a raw product, and so many times the product of diseased cows, and especially dangerous where the disease had so extended as to involve the udder, a condition found to exist in a much larger number, when carefully examined, than was generally admitted. The variety of forms which tuberculosis assumes in the human family, and the terrible losses annually occurring, made it the greatest and most important question of the day for solution, and to free our people from the dangers that lie in a polluted milk-supply must be a problem for the earliest and most thorough solution. He reviewed briefly the strong positions taken upon the subject by the leading and most advanced medical writers of the day, and favored the establishment of a veterinary sanitary police as the most crying necessity of our land in vouchsafing our people security from the dangers that are now known to be possible of control.

Dr. Gardner T. Swartes, of the State board of health, thinks that the proper officers of the State should be given the power to prevent the bringing in of diseased cattle from other States, "that all milk-animals should be tested," and that the farmer should receive ten cents per quart for milk produced under proper sanitary conditions.

